# Mechanical Stress Inference for Two Dimensional Cell Arrays

**DOI:** 10.1371/journal.pcbi.1002512

**Published:** 2012-05-17

**Authors:** Kevin K. Chiou, Lars Hufnagel, Boris I. Shraiman

**Affiliations:** 1 Department of Physics, University of California, Santa Barbara, California, United States of America; 2 Department of Cell Biology and Biophysics, European Molecular Biology Laboratory, Heidelberg, Germany; 3 Kavli Institute for Theoretical Physics, University of California, Santa Barbara, California, United States of America; Princeton University, United States of America

## Abstract

Many morphogenetic processes involve mechanical rearrangements of epithelial tissues that are driven by precisely regulated cytoskeletal forces and cell adhesion. The mechanical state of the cell and intercellular adhesion are not only the targets of regulation, but are themselves the likely signals that coordinate developmental process. Yet, because it is difficult to directly measure mechanical stress *in vivo* on sub-cellular scale, little is understood about the role of mechanics in development. Here we present an alternative approach which takes advantage of the recent progress in live imaging of morphogenetic processes and uses computational analysis of high resolution images of epithelial tissues to infer relative magnitude of forces acting within and between cells. We model intracellular stress in terms of bulk pressure and interfacial tension, allowing these parameters to vary from cell to cell and from interface to interface. Assuming that epithelial cell layers are close to mechanical equilibrium, we use the observed geometry of the two dimensional cell array to infer interfacial tensions and intracellular pressures. Here we present the mathematical formulation of the proposed Mechanical Inverse method and apply it to the analysis of epithelial cell layers observed at the onset of ventral furrow formation in the *Drosophila* embryo and in the process of hair-cell determination in the avian cochlea. The analysis reveals mechanical anisotropy in the former process and mechanical heterogeneity, correlated with cell differentiation, in the latter process. The proposed method opens a way for quantitative and detailed experimental tests of models of cell and tissue mechanics.

## Author Summary

Mechanical forces play many important roles in cell biology and animal and plant development. In contrast to inanimate matter, forces in living matter are generated by active and highly regulated processes within and between cells. The ability to directly measure forces and mechanical stress on the cellular scale within living tissues is critically important for understanding many morphogenetic processes but is a serious experimental challenge. The present work proposes an alternative approach based on the analysis of images that provide a visualization of cell boundaries in two dimensional epithelial tissues. The method uses the assumption of force balance within the epithelial layer to infer, on the basis of image-derived geometric data, the mechanical state of each cell. The proposed Mechanical Inverse method is illustrated on the analysis of two examples: the initial step of the gastrulation process in the *Drosophila* embryo, and the process of neurogenesis in the developing avian cochlea.

## Introduction

Genetics and biochemistry are central to all aspects of biological function. Physics is often less recognized yet also important at many levels, everywhere from intramolecular to organismal scales. For example, many important aspects of cell behavior depend directly and indirectly on its mechanical state defined by its interaction with neighboring cells and adhesion to the extracellular matrix [1]–[3]. Cytoskeletal mechanics and cell-cell adhesion determine geometric properties of cells [1], [4]–[6], as well as the dynamics of biological tissues [5], [7]–[13]. In plants, cells do not move, but the rigidity of cellulose membranes makes mechanical stress an obvious factor for cell division and proliferation [14], [15]. It is known that animal cell proliferation also depends on substrate adhesion and the degree of cell confinement [2], [16]–[19]. It has also been demonstrated that (stem) cell differentiation is affected by substrate rigidity [20]. More speculatively, mechanical feedback interactions have been conjectured to have a role in coordination of growth during development [1], [9], [21], [22]. Mechanical transformation of epithelial tissue is of course itself central to many morphogenetic processes: gastrulation [7] and convergent extension [1], to name a few. Understanding how mechanical changes in cells orchestrate morphological reorganization of tissues is an open problem and a subject of much current work [1], [7], [8]

Our present understanding of the role of mechanics as one of the regulatory inputs into the cell is strongly impaired by the difficulty of quantitatively characterizing the mechanical state (i.e. stress and deformation) of the cell. Among the available techniques are laser tweezers [23] and “traction force microscopy” [18], [24] performed on cultured cells. UV laser ablation allows the mechanical perturbation of tissues [8], [25], [26] on the cellular scale with the time-lapse imaging of subsequent relaxation providing information on the mechanical state of the tissue. The ablation approach is widely used on live preps, for example, in the study of Drosophila embryonic development. Yet, this technique is definitely not a “non-destructive” one.

**Figure 1:**
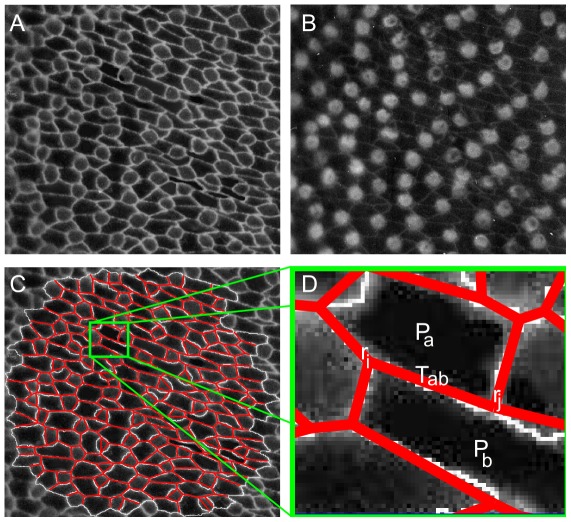
Micrographs of a fixed avian cochlear epithelium (kindly provided by Goodyear and Richardson, see [**29**] for details) at the E9 stage of development just following the onset of differentiation of cells into hair cell precursors and surrounding support cells. Panel (**A**) visualizes cell boundaries using and anti-cingulin (a tight junction protein) staning. Panel (**B**) shows the same tissue with pro-neural cells [29] stained via an anti-hair cell antigen. (**C**) is a computer generated segmentation of the raw image in (**A**) as a polygonal tiling which approximates cell geometry. The zoomed-in image (**D**) defines our parametrization of cell geometry in terms of vertex coordinates 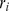 and of the mechanical state of the cell in terms of interfacial tensions 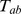 and hydrostatic pressures 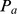.

On the other hand one of the major recent technical advances in developmental biology is the improvement of live fluorescent imaging. These provide high quality time lapse movies of developmental processes, including interesting morphological transformations such as gastrulation and convergent extension [7], [26], [27]. The purpose of the present investigation is to explore what insight into the mechanical state of cells may be gleaned from a quantitative examination of high quality images of the type shown in Fig. 1A. Our goal is to use image analysis as a non-destructive approach to obtaining quantitative measures of stress in these systems. A similar strategy has been pursued by the recently proposed “Video Force Microscopy” (VFM) approach by Brodland et al [28]. Our approach will differ from VFM in its assumptions about mechanical state of tissue, in the parameterization of forces and in the way imaging data is utilized.

Below we shall define a general model parameterizing the mechanical state of cells in two dimensional epithelial tissue and provide a computational method for inferring these parameters from the observed geometry of the cell array. We shall study the sensitivity of the proposed Mechanical Inverse (MI) method to errors in measured cell geometry and identify conditions under which robust inference is possible. We then illustrate the proposed MI method by applying it to the analysis of two different biological processes: cochlear neurogenesis [29] and ventral furrow formation[27].

## Materials and Methods

### Model of epithelial tissue mechanics

Our approach is based on the assumption that epithelial monolayers are in an instantaneous mechanical equilibrium, characterized by a static balance of the forces acting at intercellular junctions. The second important assumption is that epithelial mechanics is dominated by the actomyosin cortices and inter-cellular Adherens Junctions [1] both localized at cell boundaries, which form a visible two-dimensional web, as shown in Fig. 1A. Thus we assume that the mechanical state of a cell can be described by an effectively *two-dimensional* model with tension at the interface and the hydrostatic pressure in the cell interior. Yet, because cells can independently regulate their mechanical state, e.g. by modulating myosin activity or cell-cell adhesion, we allow for the possibility of each intercellular interface to have a different effective tension, 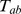, and for each cell to have a different internal pressure 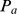 (where 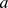 labels cells and 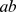 labels the interface between cells 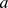 and 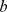), as shown in Fig. 1D. Mechanical equilibrium then corresponds to the condition that the forces acting on each “vertex” 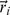 (defined as a junction of three cells and therefore of three interfaces) add up to zero.

**Figure 2:**
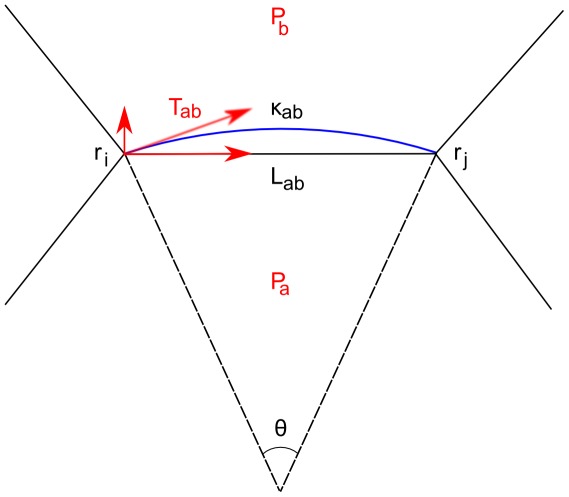
Schematic representation of an edge between two cells in the tissue, comparing a curved interface (blue) with its approximation by a chord that defines the edge in the polygonal representation of cells. Mechanical stress parameters are in red, and geometric quantities are labeled in black. Provided that the curvature of the interface 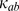 (and hence the angle 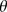) is small, the Young-Laplace equation 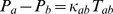 defines the force on the vertex 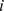 between cells 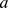 and 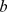 which obeys Eqn. (1).

Let 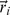 and 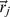 be the vertices belonging to the interface 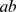 and let 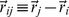 be the vector from vertex 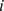 to 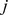. The force exerted by this interface on vertex 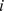 is
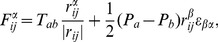
(1)where 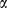 labels vector components in the 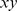 plane and 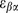 is the anti-symmetric tensor (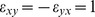 and 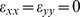). As shown in Fig. 2, this expression accurately represents the Young-Laplace balance between interfacial tension and the pressure differential across the interface 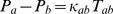, as long as the interfacial curvature 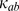 is small (see Supplementary Text S1). This fact enables us to formulate all mechanical balance conditions in terms of a polygonal approximation of the cell array, thus allowing us to reduce the problem to a generalized “vertex model” [8], .

Remarkably, the forces given by (1) correspond to the mechanical energy in the form of the following simple Hamiltonian
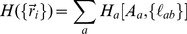
(2)where 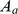 is the area of cell 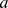, 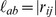 is the length of the interface between cells 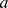 and 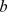 and 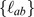 denotes the set of interfaces belonging to cell 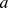. Both 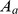 and 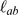
's are defined in the polygonal approximation. This Hamiltonian is a generalization of the vertex models often used to describe epithelial sheet mechanics [8], [9], [26]. Pressure and tension are defined by considering the differential form of 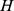:
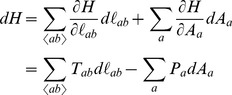
(3)where we define 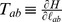 and 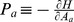. The 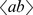 sum runs over all edges, i.e. pairs of neighboring cells 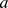, 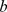. This tangent representation of mechanical energy expresses interfacial tension 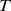 and intracellular pressure 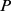 as conjugate variables to edge lengths and cell areas, respectively. (The reader will notice that strictly speaking our 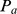 refers to a two-dimensional pressure which relates to the hydrostatic pressure only with the additional assumption that 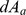 entails a change of cell volume. Alternatively 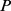 may be thought of as the axial component of the three-dimensional stress tensor.)

Mechanical equilibrium means that 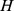 is minimized with the respect to vertex positions
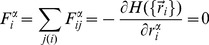
(4)which defines the static force balance constraints (the sum is over the vertices 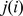 neighboring 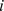). Our analysis will be based on the assumption that the cell layer is close to mechanical equilibrium in the sense of the magnitude of the net resultant force acting on vertices being much smaller than the average magnitude of the component forces that vectorially add to the resultant 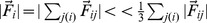. Stated in other words, we assume that the internal forces that balance each other in the (approximate) instantaneous mechanical equilibrium state are much larger than the unbalanced residual force that drives residual physical motion and (through viscous effects) defines its velocity. More generally, the dynamics of passive relaxation towards this mechanical equilibrium would be described by 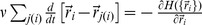, where 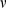 is the “effective viscosity” and the sum is again over the vertices 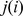 that neighbor 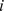. In principle, given that vertex velocities can be directly measured by time-lapse microscopy in live tissues, 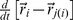 can be obtained directly from the experiment, allowing a straightforward extension of the method described below toward the VFM method [28] (where viscous forces were assumed to dominate).

### The mechanical inverse problem

**Figure 3:**
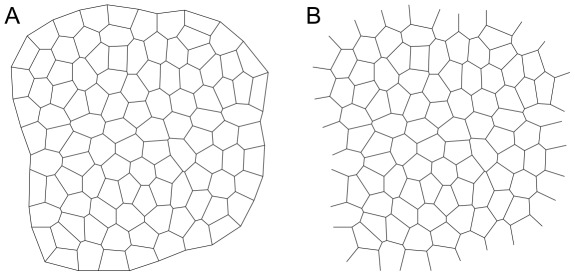
Examples of computer generated *closed* (A) and *open* (B) cell arrays. Closed arrays provide an idealized context for defining the mechanical inverse problem, while the analysis of experimental data requires dealing with open arrays, corresponding to convex patches of cells defined by or within the field of view.

We can now inquire to what extent the knowledge that a given cell array geometry is in a mechanical equilibrium constrains the parameters 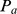, 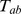 describing the mechanical state of cells. We proceed by a simple count of mechanical constraints and of the free parameters for two cases i) a closed cell array, shown in Fig. 3A and ii) an open cell array, shown in Fig. 3B.

Let us begin with the closed cell array where 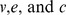 are the total number of vertices, edges, and cells, respectively. For 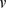 vertices in two dimensions, we have exactly 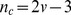 mechanical constraints, where the extra three degrees of freedom are associated with global translation and rotation symmetries (alternatively, three constraints are redundant because the total force and total torque in the closed system are equal to zero). On the other hand, the number of unknown tension parameters is 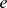, and the number of unknown pressures is 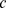, so that the total number of parameters is 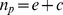. Our closed system, if we count the exterior as an additional “cell”, is topologically equivalent to a sphere so that Euler's theorem reads
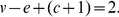
(5)Combining this relation with the condition that vertices are points where three edges meet and each edge impinges on two vertices, that is 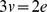, we obtain the result
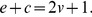
(6)This implies 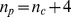, which means that our unknown parameters can be determined up to four free constants. One of the latter is the arbitrary overall scale of 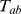 and 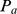 which cannot be constrained by the force balance conditions (note that since 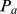 is only defined up to an additive constant, one can set the pressure in the exterior of the domain to zero). Yet the good news is that the number of free constants is finite, while the number of nontrivial constraints scales with the number of cells! This counting argument can be readily generalized (see SI) to the case where a fraction of vertices has more than three incoming edges: the so called “rosettes” that can be quite common in certain tissues [30].

Repeating the counting procedure for the open system, one finds that 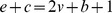, where 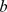 is the number of cells at the boundary of the domain. It follows that 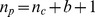. Thus mechanical parameters are determined up to 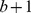 free constants: we can still choose the overall scale while the additional 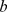 degrees of freedom may be regarded as the boundary conditions such as 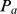
's of the cells at the edge of the domain. Again, for a large array, because 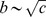 while 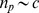, the number of parameters and constraints is much larger than the number of free constants.

To actually determine the 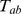 and 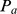 parameters, we use the fact that they appear only linearly in the force balance equations (4). This results in a linear system for
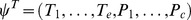
(7)in the form
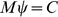
(8)with 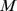 being an 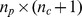 matrix where the 1st 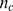 rows impose the force balance conditions from equation (4), and the additional row imposes the scale. This is performed by constraining the average tension to be equal to one. Correspondingly the top 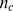 entries of the column vector 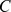 are zero, while the bottom row 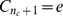.

The rectangular system (8) is solved via a pseudo-inverse [31] with the general solution of the form
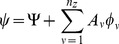
(9)with
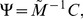
(10)

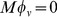
(11)where 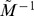 is the pseudo-inverse of the rectangular matrix 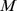 and the free parameters, 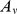, are the amplitudes of the 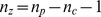 “zero modes” 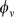. Additional details regarding the formulation and solution of the inverse problem are provided in the Supplementary Text S1.

Fixing the remaining 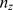 degrees of freedom requires introducing additional constraints: e.g. one may have reasons to seek a solution that minimizes variation of 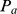
's or 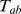
's. In choosing such additional assumptions one may want to use all the information that one has for specific applications, as we shall do below. However, before proceeding to the applications we must consider the issue of error sensitivity.

### Sensitivity of the inverse

Our approach to mechanical parameter inference is based on the observed geometry of the cell array. How sensitive are the results to the inaccuracy of vertex positions 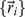
? Such inaccuracies will inevitably arise in the process of imaging, image segmentation, and more importantly from the fact that the cells themselves fluctuate. (These fluctuations are of course related to the fact that mechanical equilibrium is itself at best approximate.) To quantify the stability of the inverse we consider the effect of an arbitrary small perturbation in vertex positions, 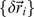. Since the inhomogeneous term 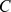 in (8) is independent of cell geometry, the first order response of the parameters 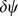 to positional error is given by
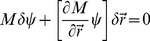
(12)

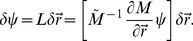
(13)


**Figure 4:**
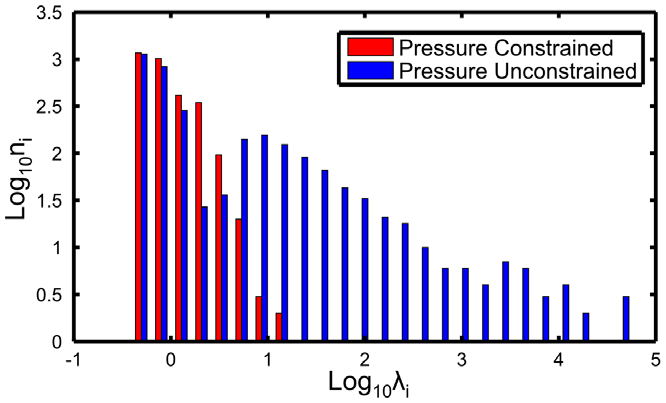
The distribution of singular values (which correspond to the square root of non-zero eigenvalues of 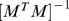) for the 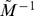 matrix before (blue) and after (red) parameter reduction. Note that prior to parameter reduction there is a substantial fraction of eigenvalues 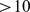 which means that small errors in vertex positions are significantly amplified in solving the inverse problem. Large eigenvalues are effectively suppressed after parameter reduction.

Ideally the error response matrix 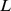 would have small eigenvalues providing a relatively robust inverse. On the other hand, large eigenvalues of 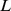 would indicate high error sensitivity. These sensitive modes appear via the pseudoinverse matrix 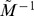. A histogram of singular values of the matrix 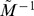 is shown in blue in Fig. 4 (for a closed system with 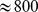 cells). One notes that a substantial fraction of modes have eigenvalues larger than one. As a result, small errors in positions can result in large error in inferred parameters.

The simplest way to solve the sensitivity problem is to reduce the number of parameters. For example, as we shall argue below, in some contexts it may be reasonable to neglect variation in cell pressure and set 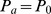 which eliminates 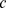 parameters, reducing 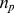 from 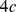 to 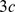. In that case the mechanical constraint system given by (8) becomes overdetermined and can be solved only in the sense of least square minimization: i.e. minimization of
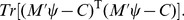
(14)The solution of the minimization problem is still given by the pseudo-inverse of the rectangular matrix 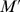 which extends the force balance matrix 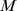 by including additional (linear) equations that constrain 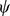. Fig. 4 shows (in red) the distribution of singular values governing the sensitivity of the reduced or *partial* inverse problem. We note a substantial reduction in sensitivity.

**Figure 5:**
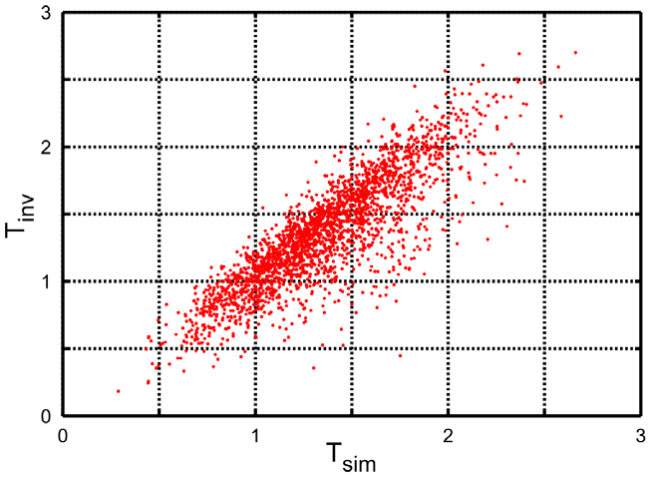
Scatter plot comparing actual values of the tension parameters 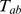 defining the *in silico* cell array to the values inferred by the partial inverse algorithm applied to the vertex data corrupted by 5% random noise. The plot exhibits a correlation coefficient of 0.85 between the estimated and actual tensions.

The partial inverse approach is then tested *in silico*. To that end we consider a closed array of cells and define cell geometry by minimizing elastic energy given by
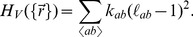
(15)with uniformly distributed 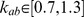. The absence of area terms imposes a constant pressure. (The closed cell array is relaxed under toroidal boundary conditions to prevent a collapse into the zero tension ground state.) The vertex model parameters are computed via equation (2). These quantities are then compared to values obtained by applying the partial inverse algorithm to the vertex “data” 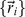 corrupted by random noise 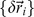 with an r.m.s. variation of 5% of the average length of cell edge (see Fig. 5). The correlation coefficient between inferred and computed parameters is 0.85, which confirms the ability of our method to extract information from noisy data. The Supplementary Figure S4 shows results under a 10% random corruption in vertex positions. In that case the correlation coefficient is reduced to 0.65, which remains serviceable.

We note that the”soft modes” that contribute to the sensitivity of the full inverse problem are quite interesting. The formulation of the minimally constrained problem is analogous to the isostatic systems studied in jamming transitions of amorphous solids [32]. These isostatic systems live on the boundary of Maxwell's criterion for rigidity, and much like amorphous solids they must satisfy both the local and global rigidity conditions. In our mechanical inverse formulation, “rigidity” corresponds to a fully constrained set of mechanical (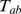 and 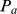) parameters. Amusingly, local soft modes for the MI problem correspond to special local geometries: specifically, polygons that can be inscribed into circles (i.e. a generalization of regular polygons) - a category which includes triangles of any shape. These interesting mathematical aspects of the problem will be discussed in a separate publication.

## Results

### Mechanical differentiation of cells in the developing avian cochlea

In cochlear development, which takes place during the first two weeks of chick embryonic development, cells in an initially homogeneous two dimensional epithelial layer differentiate into pro-neural (hair-cell) and support cell fates [29]. The process is driven by Delta/Notch-mediated cell-contact signaling [33], which causes lateral inhibition: cells which express Delta ligand on their surface prevent their immediate neighbors from doing the same. Expression of Delta is an early marker of the pro-neural fate of cells. Fig. 1A,B presents a micrograph of the cochlea epithelium, obtained by Goodyear and Richardson [29] at the stage of development shortly after the onset of differentiation. The images in Fig. 1A,B were obtained as described in [29] using a double fluorescent antibody labeling: antibody to the tight junction protein cingulin allowing visualization of cell boundaries and 275 kDa hair-cell antigen staining labeling pro-neural cells. Note that the two cell types already have discernibly different morphology: pro-neural cells are somewhat smaller and have curved edges. This dimorphism is supported by direct labeling of specific pro-neural markers, shown in Fig. 1B and demonstrated in [29].

Our goal is to infer, based on the analysis of the image in Fig. 1A, the variation in the mechanical parameters between cells. The visible positive curvature associated with pro-neural cells suggests that they are under higher internal pressure. Can the Mechanical Inverse method determine pressure differentials between cells? Because our approach requires only positions of cellular vertices, it does not use the information provided by the interfacial curvatures which are readily measurable on the image. This additional information will be used as an *a posteriori* validation of the inferred results.

**Figure 6:**
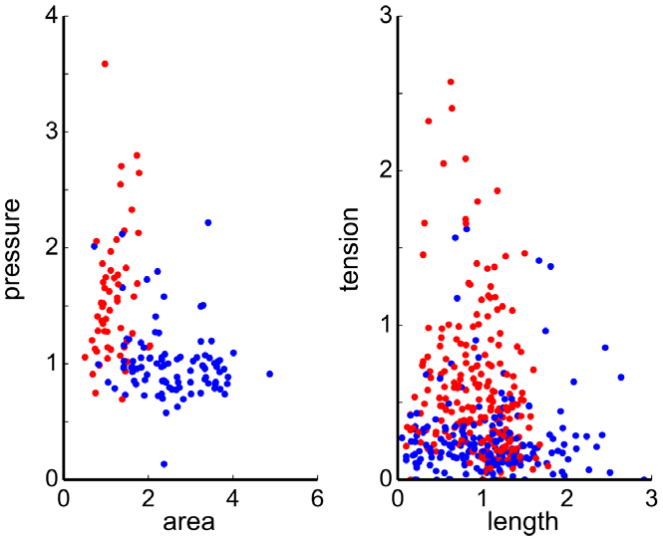
Inferred tensions and pressures for the cochlear epithelium image shown in **Fig. 1A**. Hair-cell precursors and support cells correspond to red and blue dots respectively. Inferred pressure is plotted versus the observed cell area and the inferred tension is plotted versus edge length. Note systematically higher inferred pressure and tension in the hair-cells.

**Figure 7:**
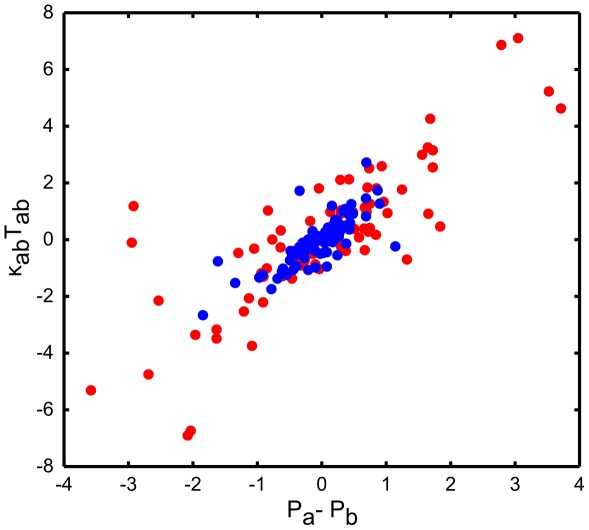
Scatter plot comparing inferred pressure differential across an interface, 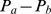, with the product of inferred tension 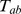 and the measured curvature 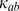 of the same interface. Different colors distinguish results obtained from different images. The scatter plot exhibits a clear correlation between the two quantities, as expected from the Laplace' law 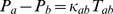.

To reduce the number of parameters, we assume that interfacial tensions can be expressed as 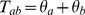 in terms of constant cortical tensions 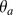, 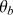 of adjacent cells which reduces the number of parameters by 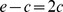. This is sufficient to render a robust partial inverse (in the sense of least squares), yielding 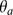 and 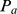 for every cell. Fig. 6 shows the distribution of inferred intracellular pressures and cortical tensions for the two cell types. We see that pro-neural cells have on average higher tension and pressure. While pressure shows some correlation with cell area, there is no correlation between interfacial tension and its length. However, there is no reason to expect any specific correlation between these quantities. On the other hand, Laplace's Law predicts 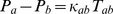 which we are in a position to check directly, thanks to the fact that interfacial curvatures 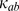 are directly measurable on the images such Fig. 1A. Fig. 7 presents the “empirical” Laplace's Law obtained on the basis of the inferred 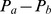 and 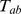. Because the Mechanical Inverse algorithm did not in any way use the interfacial curvature information, the fact that inferred parameters approximately obey the Laplace's Law provides a validation of the inverse method.

### Mechanical anisotropy at the onset of the ventral furrow formation in Drosophila

**Figure 8:**
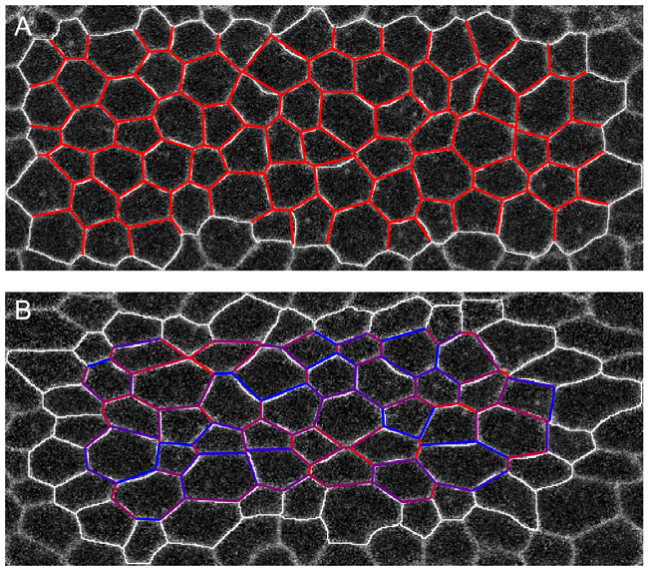
Confocal images of Spider-GFP labeled cells on the ventral side of a Drosophila embryo 4 minutes and 2 minutes prior to ventral furrow invagination [27]. (Previously unpublished images kindly provided by the Wieschaus' laboratory). Panel (**A**) shows the polygonal tiling array defined by image segmentation at 4 min prior to invagination. Panel (**B**) shows inferred tractions obtained from the partial inverse and Eq. (16) at 2 min prior to invagination. Color indicates the magnitude of inferred traction with red (blue) being the relatively high (low) traction. The coefficient of variation of inferred traction is 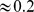.

During the initial stage of development a Drosophila embryo is comprised of an ellipsoidal monolayer of cells. The first step toward more complex morphology that is achieved after gastrulation, is the formation of a ventral furrow that begins with the contraction of the apical surfaces of cells along the ventral midline [27], [34], [35]. Fig. 8A presents the ventral view of a Drosophila embryo at the beginning of this mechanical transformation. The high quality of these images (kindly provided by the Weischaus lab [27]) makes it possible to attempt the Mechanical Inverse analysis. Since the process begins even before cellularization is completed it is reasonable to assume that cells have the same internal pressure 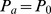, allowing us to reduce the number of parameters enough to achieve a robust partial inverse and infer 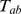 for every cell boundary. Note that in contrast to the developing avian cochlea, cell-cell interfaces exhibit little curvature and (apical surfaces of) cells are well approximated by polygons (see in Fig. 8A), which is consistent with pressure differentials being weak compared to interfacial tensions.

**Figure 9:**
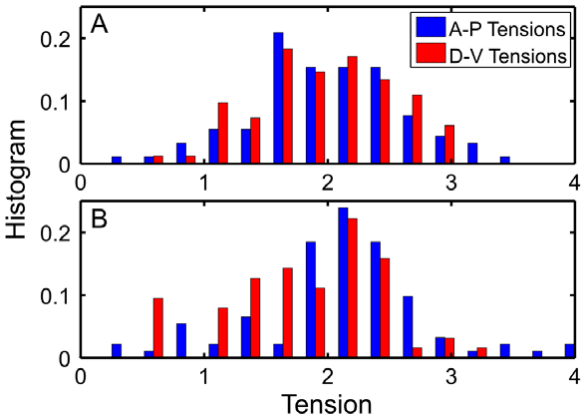
Histograms of inferred tension at the start of the ventral furrow formation. Red (blue) corresponds to cell edges at an angle above (below) 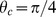 relative to the AP axis. Panels (**A**) and (**B**) correspond to respectively the 1st and the 3rd minutes of the furrow formation process.

Interestingly, comparing images separated by merely two minutes (Fig. 9) we found that the inferred 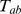 at the later time-slice exhibited statistically significant anisotropy with estimated tensions of cell interfaces along the AP axis being on average about 15% higher than those along the DV axis. The inferred increase in AP tension (relative to DV) is consistent with the laser ablation measurements made in the Wieschaus lab [7], [27]. Yet, mechanical inverse inference gives information not only on the global, tissue-wide level, but also on the scale of a single cell and interface. The analysis also clearly demonstrates the ability to make specific predictions (for interfacial tensions) that can be directly tested by combining high quality live imaging with UV pulsed laser ablation.

### Intercellular traction forces

**Figure 10:**
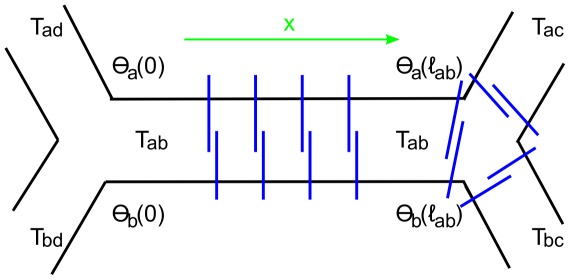
Schematic decomposition of the effective interfacial tension into cortical tensions acting within cells: 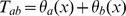. Because cytoskeletal cortexes of cells are crosslinked by cadherins via Adherence Junctions, indicated in blue, cortical stress can be transferred from one cell to another so that 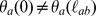. The corresponding traction force (or shear stress) is given by Eqn. (16).

The variation of tension from one interface to another implies the existence of traction forces acting between cells. This traction, or shear stress, must be entirely borne by the cadherins and other cell adhesion molecules which bridge cellular membranes and connect actomyosin cortices of apposing cells [1]. In Fig. 10 we zoom in on an interface decomposing interfacial tension into the cortical tensions on the opposite sides of the interface 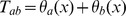, now allowing for the possibility that the latter are not constant along the interface and vary as a function of position along the edge 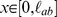. This transfer of tension from the cortical bundle in one cell to the other is possible because of cadherin mediated traction forces acting between cells. The total shear stress on the interface is 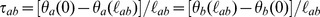. In the Supplementary Text S1 we show that because cortical tensions are constrained by the continuity conditions at cell “corners” they can be readily expressed in terms of interfacial tensions leading to the following simple expression for the traction force acting between cells 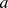 and 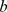.
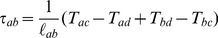
(16)
Fig. 8B shows inferred tractions calculated for the ventral furrow data taken two minutes prior to invagination. We observe a significant variability in tractions at different interfaces. Because traction forces stretch trans-cellular cadherin dimers, they may be physiologically important. Since at present there is no way of measuring them directly the possibility of indirect inference is particularly interesting.

## Discussion

We have demonstrated that the readily visualized two dimensional network of cellular interfaces in an epithelial tissue holds, potentially, a wealth of information on the relative strength of mechanical stresses acting in the tissue. The main precondition is that the tissue is close to the mechanical equilibrium in which internal cytoskeletal forces are balanced by intercellular interactions. Any imbalance of forces corresponding to directed or fluctuating motion must be small in comparison to the magnitude of internal forces that balance each other in mechanical equilibrium. Force balance is achieved by the suitable adjustment of cell geometries (parameterized by the positions of vertices). Conversely we envision changes in tissue geometry to be driven adiabatically - i.e. without disruption of the mechanical equilibrium - by changes in cytoskeletal forces within cells. This picture is at once similar and dissimilar to the case of soap froths. The geometry of a soap froth [36]–[38] is also defined by the instantaneous force balance and changes adiabatically (when gas diffuses out of cells with higher internal pressure). Yet epithelial cells, in contrast to soap bubbles, can control interfacial tension by regulating myosin activity within actomyosin cortices and therefore can generate variation in tension on sub-cellular scale, even between different interfaces of the same cell.

Our Mechanical Inverse method is fundamentally different from the Video Force Microscopy [28]. In contrast to our assumption that cytoskeletal forces are in an approximate instantaneous balance, VFM is based on the assumption that bulk forces acting within the tissue are balanced by viscosity. It is therefore based on the observed velocity of tissue motion and employs finite element methods to define forces on a computational grid rather than the underlying cells. The two methods are complementary in the sense that VFM provides information about the distribution of unbalanced bulk force which drives motion on the scale of the embryo, while our Mechanical Inverse is focused on the internal balance of forces in relation to cell geometry and its local changes. Our approach can be extended to include measured velocities which can be used to define net forces on the vertices, as explained below Eq. (4), leading to a modified inverse problem. This generalization would bridge the static inference presented here with the VFM approach. Yet, to the extent that the dynamics of normal epithelial cell rearrangement unfolds relatively slowly (on the time scale of minutes) compared to the rapid (time scale of seconds) viscosity limited retraction of laser ablated interfaces, it is reasonable to assume that the contribution of viscous forces during slow normal developmental dynamics is small compared to the balancing internal forces, which is the assumption underlying our Eqn. (4).

Recent experiments have demonstrated that actomyosin structures transiently assembling on the apical or basal surfaces of the cell, play an active role in defining its mechanical state [7], [26], [39]. In particular, [7] and [26] argue that coalescing pulses of “medial myosin” on the apical surface drive a ratchet of apical surface contraction. Presently, our mechanical model does not explicitly incorporate such effects, which in full generality would require introduction of many more parameters (characterizing intracellular heterogeneity and anisotropy). On the other hand, these effects are not observed in all epithelial tissues at all times, leaving the present approach with many possible applications. Furthermore it may be possible to generalize our approach to model medial myosin as well, especially if additional information from cell imaging is used. For example, during convergent extension investigated in [26] one often observes intracellular medial myosin filaments attaching to the lateral cortex and causing measurable deformation of cell-cell boundary. It that case it may be possible to define an additional “vertex” corresponding to the attachment point, apply considerations of mechanical balance discussed above and obtain an estimate of the force applied by the medial myosin as compared to the cortical tension. Alternatively, when medial actomyosin structures appear to be isotropic, their effect may be well approximated by a uniaxial stress which is already parameterized already by our existing model. Studying the effect of medial myosin would be an interesting direction for future work.

The proposed Mechanical Inverse method converts clearly stated assumptions about the nature of cellular stresses into readily falsifiable predictions. Using the example of avian cochlea, we were able to demonstrate that mechanical parameters inferred via the Mechanical Inverse satisfy non-trivial cross-checks provided by independent additional information (interfacial curvature measurements) read off the tissue images. Thus our approach is capable, in realistic applications, of inferring mechanical parameters and to uncover interesting aspects of the internal state of the cell. By combining high quality live imaging with UV pulsed laser ablation, one will be able to put predictions for local interfacial tensions obtained via the Mechanical Inverse, to a rigorous experimental test. We note however, that the predictions do not have to be very accurate to be useful. Even if inferred tensions each carry only a single bit of information - i.e. identify interfaces with high or low tension - correlating tension with the observed level of myosin, cadherin and/or other proteins involved in regulation of cell mechanics could be extremely informative (in addition, since a large number of cells can be imaged and analyzed, the method is effectively “high throughput”!). Finally, our approach allows for inference of quantities such as inter-cellular traction forces (or shear stress), which may be important for the stability of Adherens Junctions but cannot be directly measured by any means presently available. Future development of FRET based molecular sensors of stress [40] may nevertheless make such measurements possible in the future. Hence we expect that further development, validation, and application of the Mechanical Inverse method will lead to new insights into the molecular biology of epithelial cells and tissues.

## Supporting Information

Figure S1 A comparison of inferred tensions between two optimization schemes: linear least squares (i.e. the pseudo-inverse) and linear least squares with a tension positivity constraint (i.e. quadratic programming). Note the small tail of negative tensions predicted (by the pseudo-inverse) when positivity is not imposed.
(TIF)

Figure S2 Cumulative Distribution Functions (CDF) of inferred AP and DV tensions at the outset of *Drosophila* gastrulation, four minutes and two minutes prior to invagination of the ventral furrow. The broken (solid) lines indicate distributions obtained at the earlier (later) time step. The red (blue) lines indicate tensions oriented predominantly in the DV (AP) direction.
(TIF)

Figure S3The “collapse” of two three-fold coordinated vertices into a four-fold coordinated vertex that is shared by cells 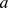, 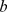, 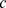, and 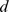 (also known as a “rosette”). This process can be performed iteratively by collapsing more three-fold coordinated vertices to obtain vertices of arbitrary order. Each “collapse” creates a new vertex that is of one coordination higher than the previous, as well as subtracting one vertex and one edge from the total count.(TIF)

Figure S4Tension inference of pressure-constrained simulated tissue under artificially induced 10% error in vertex positions. The correlation coefficient here 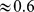. While individual tensions may deviate considerably from their simulated values, the correlation between inferred and simulated tensions is still significant.(TIF)

Figure S5 Scatter plot of edge length against mechanically inferred tension of two time points during *Drosophila* ventral furrow formation. Panel A and B respectively show results from 4 and 2 minutes prior to invagination. Analysis indicates only a very weak negative correlation between inferred tension and edge length.
(TIF)

Text S1 Additional technical details on the a) vertex model; b) computational Implementation of the Mechanical Inverse; c) interfacial traction; d) tension anisotropy; e) counting argument with higher order vertices.
(PDF)
