# Supplementary figures and images for: Mechanical Stress Inference for Two Dimensional Cell Arrays
Source: PLoS Comput Biol. 2012 May 17;8(5):e1002512. doi: 10.1371/journal.pcbi.1002512 (PMC3355066; doi:10.1371/journal.pcbi.1002512)

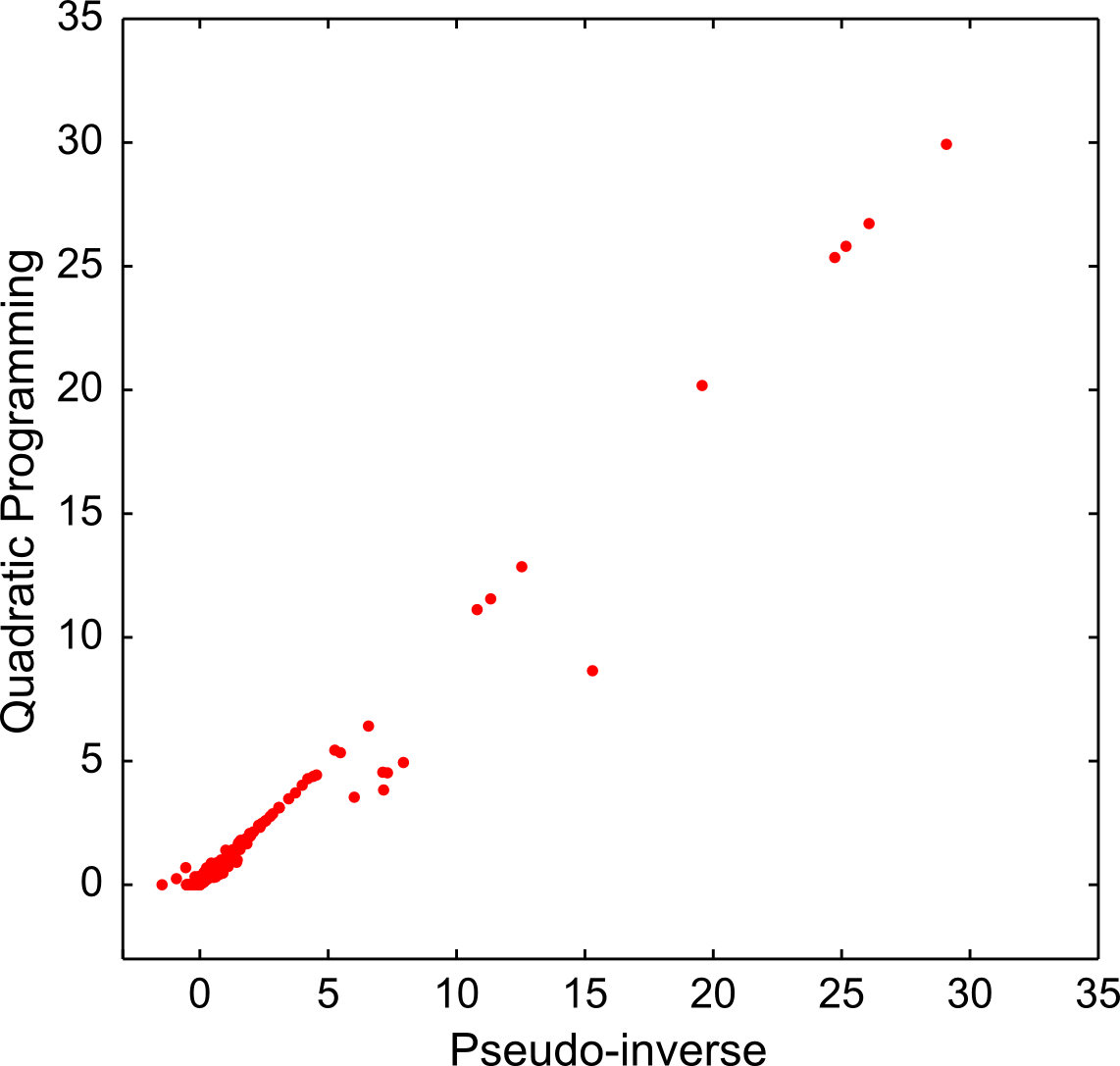

Supplement: Figure S1 — A comparison of inferred tensions between two optimization schemes: linear least squares (i.e. the pseudo-inverse) and linear least squares with a tension positivity constraint (i.e. quadratic programming). Note the small tail of negative tensions predicted (by the pseudo-inverse) when positivity is not imposed. (TIF) [file pcbi.1002512.s001.tif]

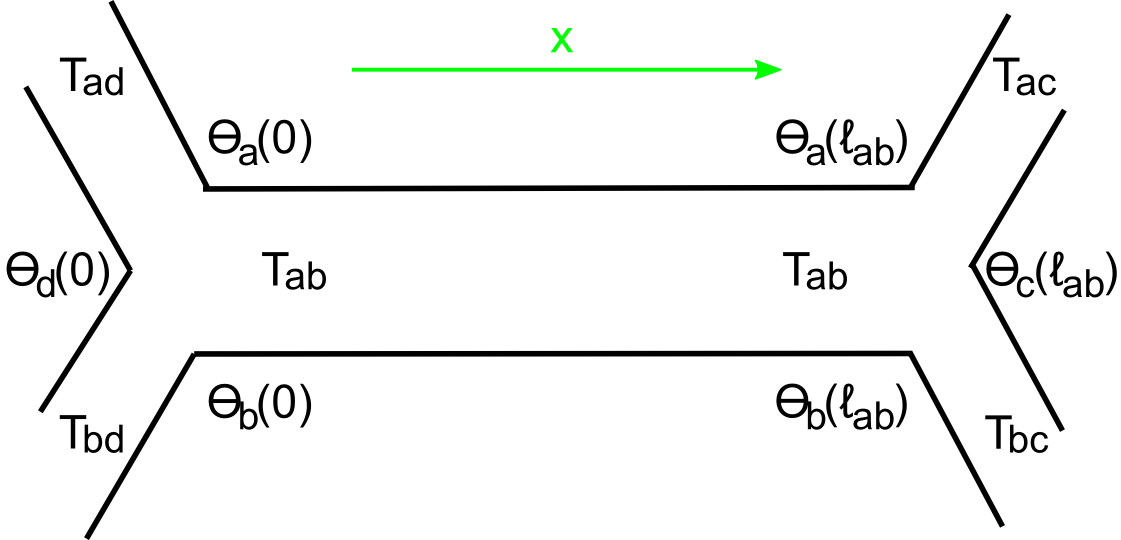

Supplement: Figure S2 — Cumulative Distribution Functions (CDF) of inferred AP and DV tensions at the outset of Drosophila gastrulation, four minutes and two minutes prior to invagination of the ventral furrow. The broken (solid) lines indicate distributions obtained at the earlier (later) time step. The red (blue) lines indicate tensions oriented predominantly in the DV (AP) direction. (TIF) [file pcbi.1002512.s002.tif]

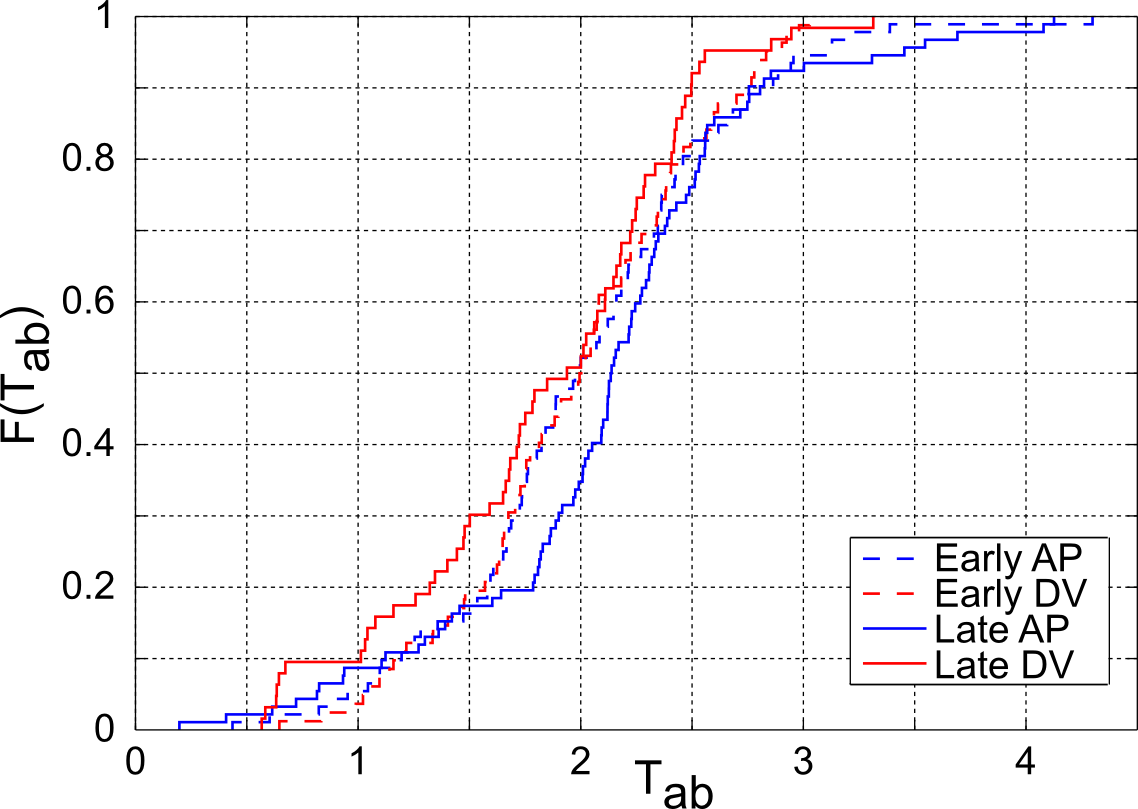

Supplement: Figure S3 — The “collapse” of two three-fold coordinated vertices into a four-fold coordinated vertex that is shared by cells , , , and (also known as a “rosette”). This process can be performed iteratively by collapsing more three-fold coordinated vertices to obtain vertices of arbitrary order. Each “collapse” creates a new vertex that is of one coordination higher than the previous, as well as subtracting one vertex and one edge from the total count. (TIF) [file pcbi.1002512.s003.tif]

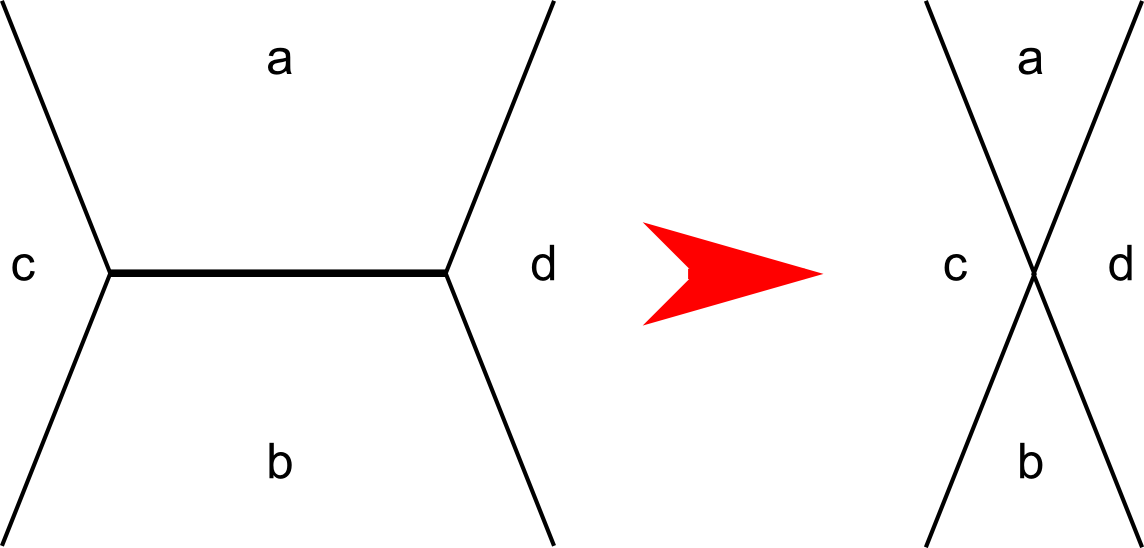

Supplement: Figure S4 — Tension inference of pressure-constrained simulated tissue under artificially induced 10% error in vertex positions. The correlation coefficient here . While individual tensions may deviate considerably from their simulated values, the correlation between inferred and simulated tensions is still significant. (TIF) [file pcbi.1002512.s004.tif]

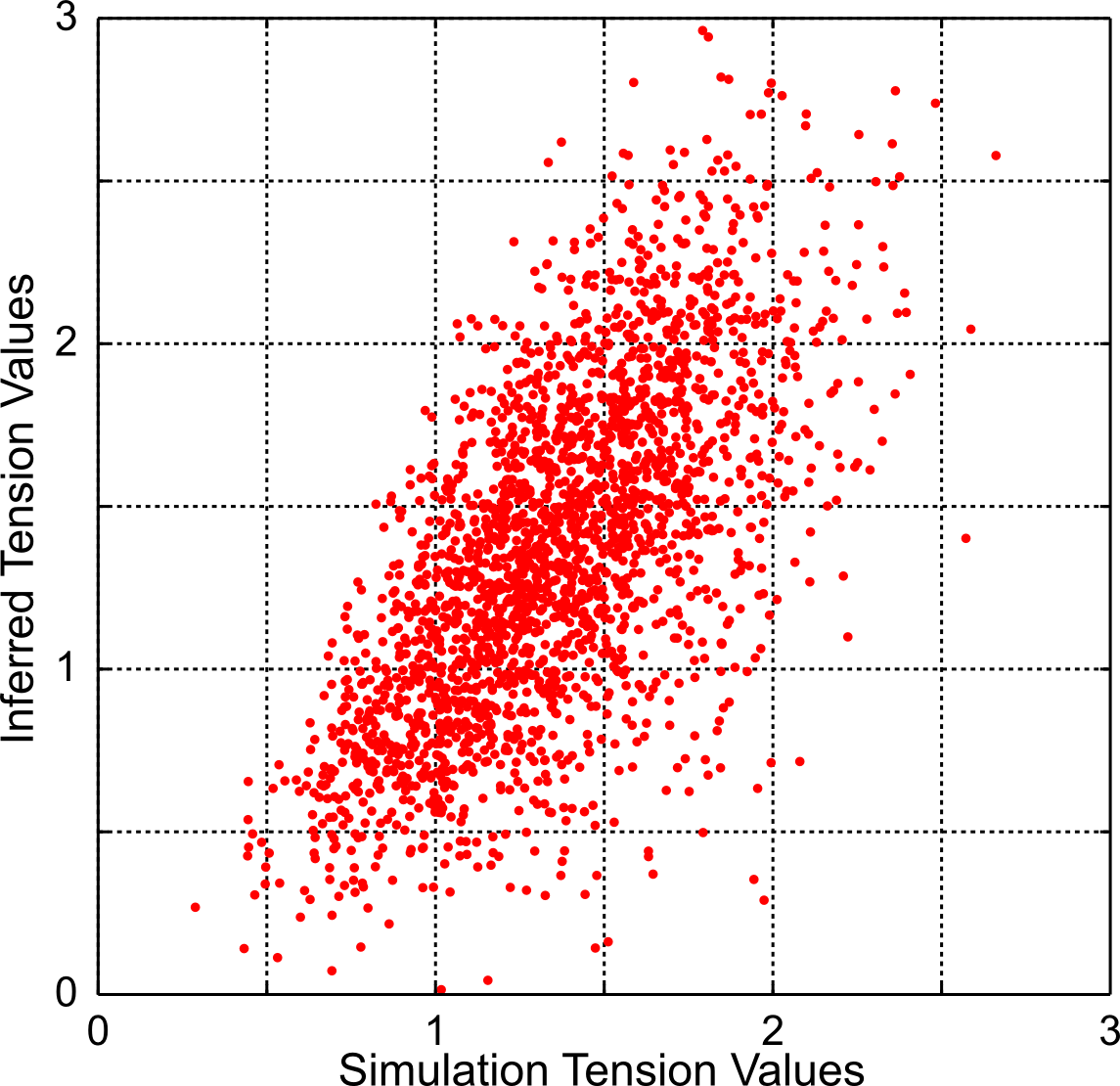

Supplement: Figure S5 — Scatter plot of edge length against mechanically inferred tension of two time points during Drosophila ventral furrow formation. Panel A and B respectively show results from 4 and 2 minutes prior to invagination. Analysis indicates only a very weak negative correlation between inferred tension and edge length. (TIF) [file pcbi.1002512.s005.tif]
