# Supplementary material for: Mechanical Stress Inference for Two Dimensional Cell Arrays
Source: PLoS Comput Biol. 2012 May 17;8(5):e1002512. doi: 10.1371/journal.pcbi.1002512 (PMC3355066; doi:10.1371/journal.pcbi.1002512)
Supplement: Text S1 — Additional technical details on the a) vertex model; b) computational Implementation of the Mechanical Inverse; c) interfacial traction; d) tension anisotropy; e) counting argument with higher order vertices. (PDF) [file pcbi.1002512.s006.pdf]

Supplementary Information for Mechanical Stress Inference for Two Dimensional Cell Arrays

Kevin K. Chiou[1], **Lars Hufnagel**[2], **Boris I. Shraiman**[1,3,*]
1 Department of Physics, University of California, Santa Barbara, CA 91306, USA.
2 Department of Cell Biology and Biophysics, European Molecular Biology Laboratory, Heidelberg, Germany.
3 Kavli Institute for Theoretical Physics, University of California, Santa Barbara, CA 91306,USA.
* E-mail: shraiman@kitp.ucsb.edu

Interfacial curvature and the vertex model. Here we flesh out the argument leading to Eq. (1) of the main text and its relation to Fig (2). Specifically, we show how a polygonal vertex model can correctly represent the effect of interfacial curvature caused by differential intracellular pressure.

Let us focus in on a cell-cell interface with tension T_{ab} in a hexagonal lattice, as in Fig. (2). The forces on a vertex due to the tension T_{ab} can be resolved into components tangential and normal to the ab-chord. If the interface has curvature κ_{ab} that spans angle $\theta \approx \kappa_{ab}\ell_{ab}$ over the length ℓ_{ab} of the interface, then the force components are

$$\vec{F} = \begin{pmatrix} F_x \\ F_y \end{pmatrix} = \begin{pmatrix} T_{ab}\cos(\theta/2) \\ T_{ab}\sin(\theta/2) \end{pmatrix}. \tag{S1}$$

If the system is in a mechanical equilibrium, the surface tension balances the difference in hydrostatic pressure on the two sides, in accordance with the Young-Laplace equation $\Delta P = P_a - P_b = T_{ab}\kappa_{ab}$. Substituting this into the y-component of the force-balance equation and taking the limit of small θ,

$$\begin{aligned} \vec{F} &= \begin{pmatrix} T_{ab}\cos(\theta/2) \\ \kappa_{ab}^{-1}\Delta P\sin(\theta/2) \end{pmatrix} \approx \begin{pmatrix} T_{ab} \\ \kappa_{ab}^{-1}\Delta P\ \theta/2 \end{pmatrix} \\ &\approx \begin{pmatrix} T_{ab} \\ \Delta P\ \ell_{ab}/2 \end{pmatrix} \end{aligned} \tag{S2}$$

where ℓ_{ab} is the length of the cord which defines cell edge in the vertex model and (for small curvatures) well approximates the original curved surface. This leads to Eq (1) where F_x component is identified with the force along $\vec{r}_{ij}$ - the direction of the "chord" connecting vertices i and j - and F_y is the force component perpendicular to the chord, i.e. in the direction $r_{ij}^{\beta}\epsilon_{\beta\alpha}$. up to a coordinate rotation.

Computational implementation of the Mechanical Inverse. Segmentation, construction of the matrix M, and inversion were all performed using custom-written Matlab programs. These programs are available upon request. Segmentation and labeling of cells is performed by the standard watershed algorithm implemented in Matlab Image Processing Toolbox. Vertex positions are obtained by identifying points of the skeletonized image that neighbor three cells.

Using cell neighbor information from the segmented image, the matrix M of Eq. (8) is constructed from Eq. (1), which represents the force on a vertex due to pressures and tensions of the neighboring cells and cell interfaces. Suppose a hypothetical cell array, obtained through image segmentation, contains v vertices, c cells, and e edges (cell-cell interfaces). Then there exist $2v$ equations

describing the vertex forces: one for each spatial dimension of each vertex. The net force vector acting on vertex i at position $\vec{r}_i$ is given in terms of neighboring vertex position vectors $\vec{r}_j$, pressures from the neighboring cells P_a and P_b, and the interfacial line tensions T_{ab}, by Eqns. (1) and (4) of the main text. The zero net force conditions correspond to the first $2v$ mechanical constraint equations.

Since all the constraints are linear in the stress parameters (T and P), determination of tensions and pressures reduces to solving a linear system of equations. However, there are two degrees of freedom that should be fixed to make the problem solvable. Since the pressure contributions only show up as differences, an overall additive pressure constant can be set. Additionally, due to the assumption of mechanical equilibrium, an overall scale must also be set. Generally we either set the exterior domain pressure is zero (closed cell arrays), or set the average pressure is zero (open cell arrays). The overall scale is set by constraining the average tension. These appear as two extra equations. For example, in the case of setting the average pressure and average tension, the equations would be of the form

$$\begin{aligned} \sum_{a=1}^{c} P_a &= 0 \\ \sum_{\langle ab \rangle} T_{ab} &= e \end{aligned} \tag{S3}$$

where a and b are cell indices, c is the total number of cells, and $\langle ab \rangle$ denotes the set of neighboring cell interfaces. Any additional constraints are incorporated via additional equations.

Once all mechanical, scaling, and additional constraints are imposed, the system is cast in the form of Eq. (8). It is then solved in the least-squares sense by way of Moore-Penrose pseudoinverse [1], which we did using the Matlab "pinv" function.

Mechanical Inversion occasionally leads to a small number of (slightly) negative tensions, as was the case in the application of the method to cochlear neurogenesis. Negative tension would correspond to an interface under compressive load. Present understanding of mechanical organization of the cytoskeletal cortex in terms of actomyosin filaments anchored to the interface by cadherin and other cell adhesion molecules, suggests that it can bear tensile but not compressive loads. (This interpretation is further supported by the fact that inferred tension is almost always positive!) We therefore modified our mechanical model by explicitly adding the constraint of positivity of tension, which adds a $T_{ab} > 0$ inequality for each edge of the cell array, complementing the system of mechanical constraint equations in Eq. (8). When mechanical constraint system is solved in the least squares sense by minimizing the quadratic form in Eq. (14), addition of a set of linear inequalities imposing tension positivity, turns it into a "quadratic programming" problem for ψ (as defined in Eq. (7)):

$$\begin{gathered} \min_{\psi} \left\{ \frac{1}{2} || M\psi - C ||^2 \right\} \\ T_i \geq 0, \forall i \in [1, \ldots, e] \end{gathered} \tag{S4}$$

which can be solved by standard quadratic programming algorithms [2]. The latter was implemented by using the Matlab routine "lsqlin" in place of "pinv". As shown in Fig. S1, imposing tension positivity via quadratic programming yields tensions very similar to the regular pseudo-inverse solution. This outcome confirms our expectation that the observed geometry of the cell array is consistent with tension/pressure model in Eq. (3).

Relating interfacial traction and cortical tension. The interfacial tension T_{ab} may be decomposed into the tension $\theta_a(x)$ carried by the cortex on the a-cell side of the interface and tension $\theta_b(x)$ carried by the cortex on the b-cell side. The two cortical components have to add up to the same total tension at each point x along the interface $T_{ab} = \theta_a(x) + \theta_b(x)$, but individual $\theta_a(x)$ and $\theta_b(x)$ can vary with x which means that tension is transferred from one side to the other. This transfer of tension is made possible by the shear stress, or equivalently, the traction force, acting at the interface of the two cells: $\tau_{ab}(x) = \partial_x \theta_a(x) = -\partial_x \theta_b(x)$. The average traction acting along the interface is

$$\tau_{ab} = \frac{1}{\ell_{ab}} \int_0^{\ell_{ab}} dx\ \tau_{ab}(x) = \frac{\theta_a(\ell_{ab}) - \theta_a(0)}{\ell_{ab}} = \frac{\theta_b(0) - \theta_b(\ell_{ab})}{\ell_{ab}} \tag{S5}$$

We shall now relate this average traction to the tensions carried by the neighboring interfaces as illustrated by Fig. 9 of the main text. Observe that since cortical tension is continuous at cell vertex junctions, then we have system of equations

$$\begin{aligned} T_{ab} &= \theta_a(\ell_{ab}) + \theta_b(\ell_{ab}) \\ T_{bc} &= \theta_c(\ell_{ab}) + \theta_b(\ell_{ab}) \\ T_{ca} &= \theta_a(\ell_{ab}) + \theta_c(\ell_{ab}) \end{aligned} \tag{S6}$$

(at vertex abc) and similarly

$$\begin{aligned} T_{ab} &= \theta_a(0) + \theta_b(0) \\ T_{bd} &= \theta_d(0) + \theta_b(0) \\ T_{da} &= \theta_a(0) + \theta_d(0) \end{aligned} \tag{S7}$$

(at vertex abd). These equations can be rewritten in terms of θ_a resulting in

$$\begin{aligned} 2\theta_a(\ell_{ab}) &= T_{ab} - \theta_b(\ell_{ab}) + T_{ca} - \theta_c(\ell_{ab}) = T_{ab} + T_{ca} - T_{bc} \\ 2\theta_a(0) &= T_{ab} - \theta_b(0) + T_{da} - \theta_d(0) = T_{ab} + T_{da} - T_{bd}. \end{aligned} \tag{S8}$$

from which it follows that

$$\tau_{ab}\ell_{ab} = \theta_a(\ell_{ab}) - \theta_a(0) \quad = \quad \frac{1}{2}(T_{ca} - T_{da} + T_{bd} - T_{bc}) \tag{S9}$$

leading to Eqn. (16) of the main text for the traction force acting between cells a and b.

Inferred tension anisotropy at the outset of Drosophila gastrulation. The pressure-constrained mechanical inverse was performed on live images of *Drosophila* ventral furrow formation from the Wiechaus lab [3]. The tensions T_{ab} were classified as anterior-posterior (AP) and dorsal-ventral (DV) depending on the angle $0 < |\phi_{ab}| < \pi/2$ the interface makes in relation to the anterior-posterior axis of the embryo. Tensions with $|\phi_{ab}| < \pi/4$ were classified as AP, and $|\phi_{ab}| > \pi/4$ - DV. The two-sample Kolmogorov-Smirnov (KS) test was used to compare the inferred AP and DV tensions at two time points, four and two minutes prior to invagination of the ventral furrow.

Figure S2 shows the cumulative distribution functions $F(T_{ab})$ of tensions at each of the two time points for AP and DV tensions. Comparisons of DV and AP tension distributions at the earlier time frame give a KS test statistic with p-value of 0.99, which supports the null hypothesis that the AP and DV tensions are drawn from the same underlying distribution. Comparisons made between the DV and AP tensions at the later time point give a KS test statistic with p-value of 0.007, which supports the alternative hypothesis that the tensions

are drawn from different underlying distributions. In particular, the our result sueggests that tensions in the AP direction are on average 15% higher than the DV direction, but with both AP and DV directions exhibit comparable and large coefficients of variation (≈ 0.33 for both distributions).

Counting for cell arrays that include higher order vertices. Note that for $n \geq 4$, an n-fold coordinated vertex can be thought of as a coalescence of $(n-2)$ neighboring three-fold vertices into a single vertex. Each coalescence results in a graph with $(n-3)$ fewer vertices and edges. The lowest order example of this process can be seen in Fig. S3. Using this intuition, we formulate a counting scheme that closely follows the case analyzed in the main manuscript.

Consider a two-dimensional cell array (and therefore planar graph) g where there exist $\{v_n : n \in \mathbb{N}, n \geq 3\}$ vertices that are n-fold coordinated. Let v and e be the total number of vertices and edges in this cell array. Now allow us to define some new non-unique "effective" graph g', where g' has the following properties: all the vertices in this graph are three-fold coordinated, and by collapsing some subset of these vertices together, we recover the original cell array g. Then if v_{eff} and e_{eff} are the number of vertices and edges respectively in this graph g', they satisfy the relation

$$3v_{\text{eff}} = 2b_{\text{eff}} \tag{S10}$$

as before. These quantities are then related to v and e in the original graph g by

$$\begin{aligned} v &= v_{\text{eff}} - \sum_{n=4}^{N}(n-3)v_n \\ e &= e_{\text{eff}} - \sum_{n=4}^{N}(n-3)v_n. \end{aligned} \tag{S11}$$

Putting this together with Eq. (S10), we see that

$$3v = 2b - \left(\sum_{n=4}^{N}(n-3)v_n\right) \tag{S12}$$

is the relation for cell array g. Applying the Euler theorem to the graph g, we see that for a closed array the relation between vertices, edges, and cells is

$$2v - e - c = -1 - \sum_{n=4}^{N}(n-3)v_n. \tag{S13}$$

The calculation is similar for the open cell array. Equation (S13) dictates that each n-fold vertex adds $(n-3)$ degrees of freedom to the Mechanical Inverse. These degrees of freedom come about in a way similar to boundary cells in the open array. Boundary cells can be thought of as cells where edges and vertices are also subtracted off from some "effective" closed array. As a result, stability of the partial inverse is preserved as long as the number of new degrees of freedom remain small in comparison to the number of additional constraints imposed.

References

[1] Ben-Israel A, Greville TNE (2003) Generalized Inverses: Theory and Application. CMS books in mathematics. New York: Springer, 420 pp.

[2] Nocedal J, Wright SJ (2006) Numerical Optimization. Ann Arbor: Springer, 706 pp.

[3] Martin AC, Gelbart M, Fernandez-Gonzalez R, Kaschube M, Wieschaus EF (2010) Integration of contractile forces during tissue invagination. J Cell Biol 188: 735–749.
